# Chaperones and Beyond as Key Players in Pluripotency Maintenance

**DOI:** 10.3389/fcell.2019.00150

**Published:** 2019-08-02

**Authors:** Camila Felix de Lima Fernandes, Rebeca Piatniczka Iglesia, Maria Isabel Melo-Escobar, Mariana Brandão Prado, Marilene Hohmuth Lopes

**Affiliations:** Department of Cell and Developmental Biology, Institute of Biomedical Sciences, University of São Paulo, São Paulo, Brazil

**Keywords:** pluripotency, chaperone, stem cells, histone, stemness

## Abstract

Pluripotency is orchestrated by distinct players and chaperones and their partners have emerged as pivotal molecules in proteostasis control to maintain stemness. The proteostasis network consists of diverse interconnected pathways that function dynamically according to the needs of the cell to quality control and maintain protein homeostasis. The proteostasis machinery of pluripotent stem cells (PSCs) is finely adjusted in response to distinct stimuli during cell fate commitment to determine successful organism development. Growing evidence has shown different classes of chaperones regulating crucial cellular processes in PSCs. Histones chaperones promote proper nucleosome assembly and modulate the epigenetic regulation of factors involved in PSCs’ rapid turnover from pluripotency to differentiation. The life cycle of pluripotency proteins from synthesis and folding, transport and degradation is finely regulated by chaperones and co-factors either to maintain the stemness status or to cell fate commitment. Here, we summarize current knowledge of the chaperone network that govern stemness and present the versatile role of chaperones in stem cells resilience. Elucidation of the intricate regulation of pluripotency, dissecting in detail molecular determinants and drivers, is fundamental to understanding the properties of stem cells in order to provide a reliable foundation for biomedical research and regenerative medicine.

## Introduction

Pluripotency is an important and unique feature attributed to specific types of cells, and can be defined as the ability of cells to replicate indefinitely in the absence of senescence (self-renewal) while retaining the differentiation potential, or the ability to differentiate into all cells of an organism (Martello and Smith, 2014). Embryonic stem cells (ESCs) are classified as pluripotent stem cells (PSCs), and represent great possibilities for research and cell therapy. ESCs can be obtained from the inner cell mass (ICM) of preimplantation blastocysts. The establishment of cultures of ESCs *in vitro* (Evans and Kaufman, 1981; Martin, 1981; Martello and Smith, 2014) brought about unquestionable advances in scientific research, as the starting point for several works that sought to explore the molecular mechanisms that maintain pluripotency. In 2006, a state of ESC-like, achieved from the reprogramming of differentiated adult cells was described, referred to as induced pluripotent stem cells (iPSCs). Reprogramming of the cells was possible through the induction of specific transcription factors (TFs), OCT4, SOX2, c-MYC, and KLF4 (Takahashi and Yamanaka, 2006). OCT4, NANOG, and SOX2 are considered key factors for the maintenance of PSCs *in vivo* and *in vitro*, forming a pluripotency core that, with additional TF and cofactors, regulates pluripotency in an expanded transcriptional network (Wang et al., 2006; Kim et al., 2008).

Although many studies have been conducted to understand the exact mechanisms by which the TFs and additional factors regulate pluripotency, much remains to be elucidated. Studies are still being conducted to understand TFs individual and integrated functioning. By unmasking pluripotency mechanisms, it will be possible to use PSCs more safely and harness their therapeutic potential, also serving as a model to understand early development, important cellular processes and diseases. Besides transcriptional regulation, other mechanisms are being recently discussed as relevant to understanding the maintenance of pluripotency in stem cells, such as chromatin conformation and proteome quality control assisted by molecular chaperones. In this review we discuss aspects of PSCs maintenance, such as TFs regulation and chromatin conformation in PSCs, as well as the relationship of chaperones, co-chaperones and ubiquitin-proteasome system (UPS) with the control of TF levels and pluripotency in PSCs. A comprehensive and integrated understanding of the events – from transcription, translation to post-translational processes – that govern pluripotency is needed to answer questions that remain unanswered in the field of PSCs.

## Pluripotency Maintenance Mechanisms

Pluripotent stem cells are regulated by a series of interconnected cellular processes that pass through the transcription, translation, and final destination of proteins through different post-translational modifications. The state of chromatin conformation is important for the exposure or concealment of regulatory regions in DNA. Regulatory regions are possible targets of several TFs, that will associate in a specific way in the DNA molecule and regulate the transcription of several genes.

Several studies described the essentiality of the TFs OCT4, SOX2, and NANOG to pluripotency. These TFs form an interconnected self-regulating core, cooperatively associating with their own promoters and co-occupying more than 300 targets in an integrated manner, finely regulating their own and also their targets expression, repressing differentiation genes and activating pluripotency (Boyer et al., 2005; Wang et al., 2006). Other TFs (referred hereafter as expanded core) associated to the main pluripotency core were described, such as STAT3, SMAD1, DAX1, REX1, ZPF281, among others (Kim et al., 2008; Bharathan et al., 2017; Collier et al., 2017; Trusler et al., 2018). In 2006, the publication of Takahashi and Yamanaka’s (2006) landmark work, describing the acquiring of PSCs from the induction of four major TFs – OCT4, SOX2, c-MYC, and KLF4 – profoundly impacted stem cell research, allowing the field of development to dispose of a new and effective tool for pluripotency studies.

Although pluripotency is controlled by the expression of a network of TFs, the levels of this expression must be highly regulated in order to maintain the pluripotent state. PSCs balance between self-renewal and differentiation potential. Some researches (briefly reviewed in Torres-Padilla and Chambers, 2014), have already shown that the levels of TFs associated with pluripotency may vary in ESCs. Even in cells of the same colony, the expression of certain factors can be heterogeneous and transient. In addition to transcriptional regulation, the levels of TFs can be modulated according to translational and degradation rates and post-translational modifications.

In order to maintain PSCs undifferentiated, a cytokine member of the IL6 superfamily, named leukemia inhibitory factor (LIF), is used in cell culture (Nicola and Babon, 2015). Briefly, in the signal transduction cascade, LIF couples with gp130 receptors and activates JAKs in the cell interior which, in turn, will activate STAT3 through phosphorylation (Huang et al., 2017). Although LIF is not essential for the maintenance of these cells *in vivo* (Stewart et al., 1992), and is not solely responsible for the maintenance of pluripotency and self-renewal *in vitro*, it is an important tool in the culture of ESCs and iPSCs. Later in this review we will discuss how STAT3 relates to an important protein complex of chaperones and co-chaperones, acting in the maintenance of pluripotency in stem cells *in vitro*.

Interestingly, the existence of different statuses of pluripotency has been reported, naïve and primed, with great variety in transcriptional and epigenetic profile (reviewed in Nichols and Smith, 2009; Hackett and Azim Surani, 2014). The primed state, as the name suggests, is a state more prone to differentiation when compared to the naïve state (Marks et al., 2012), although in both states the cells remain expressing pluripotency core TFs. In addition to these classically established states, the existence of other levels for pluripotency has recently been hypothesized. As recently proposed by Smith, pluripotency can be seen as a progression through very early different developmental stages. The author emphasizes the need for a formative pluripotency state between the naïve and primed, in which cells acquire abilities to change their genomic and epigenetic profile to proceed in the course of cell-fate commitment (Smith, 2017). While some authors defend great transcriptional heterogeneity between primed and naïve states (Marks et al., 2012), other discuss that, although the two populations have their specific transcriptional signatures, this heterogeneity is expressed in low levels between the states (Messmer et al., 2019). Much still needs to be studied in order to establish a response to these controversies. Studies exploring these states *in vivo* may contribute to the understanding of their existence as part of the development of organisms, or as artifacts of cell culture.

**FIGURE 1:**
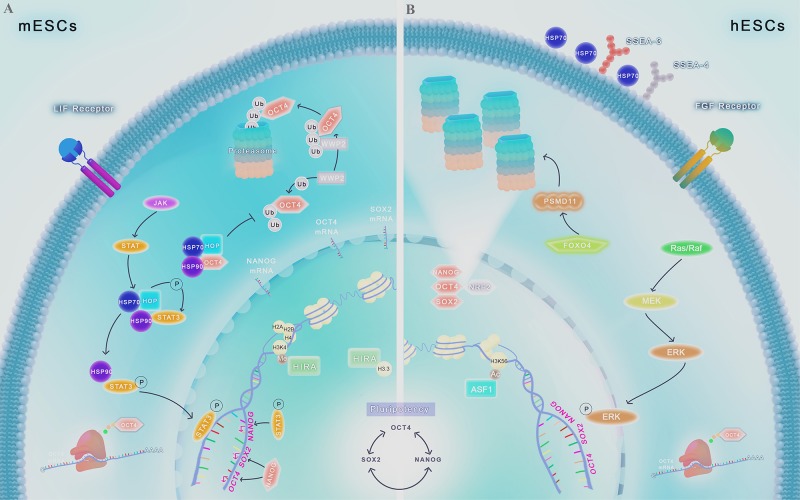
Chaperome regulation and proteostasis network in ESCs. Scheme shows molecular pathways ranging from gene transcription to protein degradation involved in pluripotency control. The interconnected self-regulating nuclear core formed by OCT4, SOX2, and NANOG is essential for the maintenance of stemness. **(A)** In mESCs, HIRA is abundantly associated with promoter regions of developmentally regulated genes, being responsible for H3.3 deposition and enrichment, co-localizing with the transcriptional active form of methylated H3K4. Chaperone protein HSP90 and its partner HOP are engaged in key intracellular signaling pathways in PSCs, including LIF/JAK/STAT3. HSP90-HOP complex participates actively in the phosphorylation and translocation of STAT3 to the nucleus, leading to the transcription of pluripotency core factors. HSPs complexes can also prevent OCT4 degradation by proteasome. Proteasome-related proteins, such as WWP2, acting as E3 ligases or by other mechanisms, lead to TFs degradation by UPS, controlling its levels and maintaining proteostasis balance in these cells. **(B)** In hESCs, FGF2, used to culture these cells, activate the signaling cascade mediated by Ras/MEK/ERK and p-ERK translocation to the nucleus, favoring the expression of pluripotency genes. Acetylation of H3K56 by ASF1 regulates de expression of pluripotency genes. Unlike differentiated cells, HSP70 is present in the cell surface of hESCs, colocalizing with known pluripotency markers such as SSEA3 and SSEA4. Upregulation of the protein FOXO4 leads to the increase of the 19S proteasome subunit PSMD11, resulting in more functional proteasome subunits formed and increased activity of the UPS. The TF NRF2 upregulation is also associated with the increase in functional proteasome subunits, and also is associated with expression of the pluripotency TFs OCT4, SOX2, and NANOG.

**FIGURE 2:**
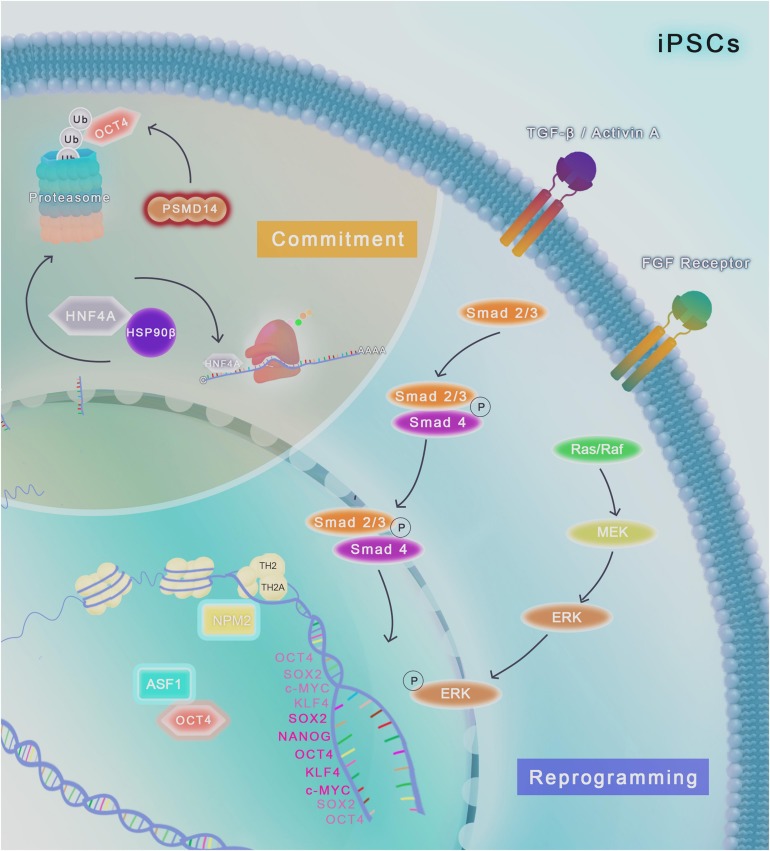
Chaperome regulation and proteostasis network in human iPSCs. TGF-β/Activin A and FGF2/Ras/MEK/ERK pathways are required for the maintenance of iPSCs in culture conditions. The histone chaperone NPM2 binds to the histone variants TH2A and TH2 and improve the reprogramming of human fibroblast into iPSCs modulated by OCT4, SOX2, KLF4 and c-MYC, generating more naïve human iPSCs compared to factors induction alone. ASF1a histone chaperone upregulation, together with OCT4, also has an important role in reprogramming of human fibroblast. Cell fate commitment (highlighted in light brown) involves the induction of different specific pathways that can lead to differentiation into various cell types. The molecular chaperone HSP90β physically binds to HNF4A and control the protein turnover of these client, modulating differentiation of iPSCs to endoderm-derived hepatic progenitor cells. Downregulation (represented as a red glow around the molecule) of the proteasome-related protein PSMD14, a 26S proteasome subunit, impairs the deubiquitylation of OCT4, leading to its degradation in the proteasome and impairment of pluripotency.

Pluripotent stem cells require elevated protein synthesis for continuous replication and thus, enhanced mechanisms of proteome quality control like elevated chaperone and proteasome activities is essential to avoid senescence and maintain stemness. The viability of stem cells critically depends on the ability to maintain protein homeostasis to adapt continuously the cellular proteome to extrinsic and intrinsic variations. The capacity of stem cells to sense and respond to changing conditions and stress is critical for normal cell growth, development and organism viability. The complexity of the proteome requires interconnected quality-control processes to meet the dynamic needs of the cell. The protein homeostasis (proteostasis) network (PN) ensures the balance of the proteome by coordinating protein synthesis, folding and conformational maintenance; and protein degradation. PN is achieved by an orchestrated system of proteins, including molecular chaperones and their regulators, which help proteins to reach its functionally active conformation, without being part of its final structure. In addition, the UPS exerts a post-transcriptional control on the levels of proteins, such as TFs, which is important to pluripotency maintenance (Figures 1, 2; Okita and Nakayama, 2012).

Considering the fine mechanics of chromatin conformation control, the importance of PN for the maintenance of cellular functions, both in health and in diseases, the increased expression of PN elements in PSCs, as well as an increased activity of PN in these cells, many studies have been conducted to understand the control of pluripotency from the perspective of these events. Tables 1, 2 summarize different molecules, addressed in this review, involved in the pluripotency control.

**TABLE 1:** Major classes of histone chaperones and their function in stemness of different PSCs models.

**Chaperone**		**Function in PSCs biology**	**References**
HIRA		Highly expressed in the promoters of developmentally regulated genes in mESCs	Goldberg et al., 2010
		Differentiation of mESCs in hemogenic endothelium	Banaszynski et al., 2013; Scambler et al., 2015
		Pluripotency maintenance of hESCs, promoting isocitrate dehydrogenase genes (IDHs) transcription	Zhu et al., 2017
		Developmental reprogramming – deposition of paternal core histone and reactivation of maternal genome in mice	Lin et al., 2014
DAXX/ATX		Telomeric deposition (immortalization) in mESCs	Elsässer et al., 2015
ASF1		Differentiation during murine early embryogenesis and gonad development	Messiaen et al., 2016
		Pluripotency maintenance in hESCs	Gonzalez-Muñoz et al., 2014
		Pluripotency maintenance in mESCs	Tan et al., 2013
		Reprogramming of human fibroblasts into iPSCs	Gonzalez-Muñoz et al., 2014
CAF-1		Early developmental arrest and early gastrulation of mESCs	Filipescu et al., 2013; Akiyama et al., 2011; Houlard et al., 2006; Hatanaka et al., 2015
		Reprogramming of mouse fibroblasts into iPSCs	Cheloufi et al., 2015
		Pluripotency maintenance during blastomeric stage in mice	Yankulov, 2015
FACT		Proliferation and neural differentiation of mESCs	Mylonas and Tessarz, 2018
		Associates with OCT4 and regulates mESCs pluripotency Survival during early blastocyst stage of mESCs	Cao et al., 2003; Ding et al., 2012; Gaspar-Maia et al., 2009; Pardo et al., 2010
		Reprogramming into iPSCs	Shakya et al., 2015; Shen et al., 2018
HMGA2		mESCs specific DNA repair mechanism.	Yu et al., 2014
NPM2		Reprogramming of human fibroblast into iPSCs; Improvement of murine cells reprogramming using only KLF4 and OCT4	Fernández-Rivero et al., 2016; Shinagawa et al., 2014
NPM3		Proliferation of mESCs	Motoi et al., 2008
SPT6		Pluripotency maintenance of mESCs	Robert, 2017
SET	SETα	Proliferation of hESCs	Edupuganti et al., 2017
	SETβSETP	Differentiation of hESCs	

**TABLE 2:** List of chaperones and proteasome-related proteins and their function associated to protein homeostasis and pluripotency control in different PSCs models.

**Proteins and families**		**Function in PSCs biology**	**References**
Chaperome	HSP90	Pluripotency maintenance and mesoderm differentiation of mESCs	Bradley et al., 2012
		STAT3 translocation to nucleus and NANOG negative regulation in mESC	Setati et al., 2010
		Endoderm differentiation of iPSCs	Jing et al., 2017
	HOP	STAT3 expression and phosphorylation and NANOG expression in mESCs	Longshaw et al., 2009
		Murine embryonic survival	Beraldo et al., 2013
	HSP70	Surface marker of pluripotency in hESCs	Alekseenko et al., 2012
		Differentiation of mESCs	Baharvand et al., 2008; Battersby et al., 2007; Saretzki et al., 2007
		Differentiation and survival of iPSCs	Brodarac et al., 2015
		Early differentiation of hESCs and mESCs	Park et al., 2011
	HSP60	OCT4 expression, proliferation, self-renewal and survival of mESCs	Seo et al., 2018
	HSP40	mESCs differentiation into smooth muscle cells Endoderm differentiation marker	Wong et al., 2014; Wang and Gudas, 1988
	HSP27	NANOG inactivation and neuronal differentiation of human placenta-derived cells	Cheng et al., 2016
Proteasome related	PSMD14	OCT4 regulation in mESCs and iPSCs	Buckley et al., 2012
	FBXW7	Negative regulation of c-MYC protein stability in mESCs and iPSCs	
	NRF2	Pluripotency maintenance in hESCs and iPSCs	Jang et al., 2014
	PSMD11	Functional proteasome complexes formation in hESC and iPSC	Vilchez et al., 2012
	F0X04	PSMD11 expression regulator in hESCs and iPSC	
	L1TD1	Downregulation leads to decrease in SOX2 and PSMD11 of hESC	Emani et al., 2015
	C0PS2	NANOG protein stability regulator of mESCs	Zhang et al., 2016
	WWP2	Promotes OCT4 and SOX2 proteasome degradation in mESCs	Xu et al., 2004, 2009, Fang et al., 2014
	SET7	SOX2 methylation and proteasome degradation promotion in mESCs Transcriptional activity inhibition in mESCs	Fang et al., 2014
	AKT1	SOX2 phosphorylation and proteasome degradation prevention in mESCs	
	UBR5	Proteostasis machinery regulator in hESCs and iPSCs	Koyuncu et al., 2018
	FBXW8	Polyubiquitynates NANOG and mESCs	Kim et al., 2014
	USP21	NANOG protein stabilization in mESCs and hESCs	Jin et al., 2016; Liu et al., 2017
	USP26	NANOG and SOX2 genes inhibition in hESCs	Ning et al., 2017

## TFs Core Regulation by Ubiquitin-Proteasome System

Regardless of the mediation of chaperones, an important fraction of polypeptides usually exhibit errors in folding or refolding (Schubert et al., 2000); consequently, they are identified and disposed by proteolytic degradation, to avoid accumulation of potentially toxic aggregate species. One major protein degradation pathway is the UPS. The UPS performs coordinated activities of enzymes that conjugate the polypeptide co-factor, ubiquitin, to proteins and tags them for degradation by an ATP-dependent process that involves three enzymes, E1 (Ub-activating enzyme), E2s (Ub-carrier or conjugating proteins) and the key E3-ligases (Ub-protein ligase) (Schubert et al., 2000; Lecker et al., 2006). The labeled proteins are identified by the 26S proteasome, which degrades them to small peptides (Lecker et al., 2006).

The protein levels of pluripotency TFs must be finely regulated for the maintenance of PSCs specific properties. It has been shown that both downregulation and upregulation beyond the required levels of some TFs, such as OCT4 (Rodriguez et al., 2008; Zafarana et al., 2009), leads to differentiation in specific tissues or impairment in stem cell identity. The UPS is one of the main post-translational mechanisms for regulating the levels of these proteins. The selectivity of proteins and aggregations for ubiquitin system presents a relevant participation of chaperones and co-chaperones, more specifically from the HSP70 family, for example HSC70 and the co-chaperone BAG3 (Arndt et al., 2010). In this section, we highlight classical and recent findings that explore the role of UPS in the control of TFs levels, essential for PSCs maintenance.

Investigating the total profile of ubiquitinated proteins in mESCs and iPSCs, Buckley et al. (2012) revealed that NANOG and OCT4 are regularly ubiquitinated, with the levels of this modification varying throughout the self-renewal process, a phenomenon that is not observed in differentiated cells (Buckley et al., 2012). The work demonstrates how these TFs are finely regulated for the maintenance of pluripotency properties in these cells, and suggests the great importance of UPS for self-renewal. It is known that elevated proteasome activity is somehow essential not only for the control of TFs levels, but also for expression of genes associated with pluripotency, cell proliferation and cell cycle progression. Defects in the proteasome leads to malfunction of all of the above processes, including a G2/M arrest in hESCs and iPSCs (Jang et al., 2014). Several research groups have been exploring the specific effects of UPS-associated proteins on the regulation of TFs in PSCs.

Upon the downregulation of PSMD14, a 26S proteasome non-ATPase subunit, the loss of OCT4 expression is observed, which is linked to an apparent dysfunction of the deubiquitinating enzymatic activity of PSMD14 (Figure 2; Buckley et al., 2012). Further, although the modulation of the E3 ligase protein FBXW7 has no direct effects on OCT4, NANOG and SOX2 expression, it has a negative effect on the protein stability of c-MYC, an important factor linked to the differentiation potential in PSCs (Buckley et al., 2012). NRF2 is a TF whose activation leads to increased levels of several proteasome subunits. NRF2 activity is increased in hESCs (Figure 1B) and iPSCs and loss of this activity results in reduced levels of OCT4, SOX2, and NANOG and also impairs proliferation, indicating a role of this protein in self-renewal (Figure 1B; Jang et al., 2014). Additionally, NRF2 colocalizes with the mentioned core TFs (e.g., OCT4, SOX2, NANOG) during differentiation, and its activation by pharmacological or molecular techniques prevents their degradation during differentiation process in hESCs (Jang et al., 2014).

The proteasome activity is enhanced in hESCs and iPSCs, with a loss of activity being observed as the cells differentiate, with a concomitant increase in differentiation factors, such as PAX6, as well as a decrease in the expression of pluripotency TFs (Vilchez et al., 2012). Furthermore, the transcription factor FOXO4 has been shown to be an important modulator of proteasome activity in hESCs, since it regulates the expression PSMD11, a 19S proteasome subunit. The increased expression of PSMD11 is sufficient to increase the number of functional proteasome complexes formed, increasing proteasomal activity (Figure 1B; Vilchez et al., 2012). Interestingly, PSMD11 levels are somehow related with other pluripotency proteins. Decreased levels of L1TD1, an RNA binding protein, correlates with decreased levels of PSMD11 (Emani et al., 2015). Pharmacological inhibition of proteasome activity leads to decrease in L1TD1 and SOX2 in hESCs (Emani et al., 2015). However, the exact mechanisms by which these molecules interact with each other, and how they interact with OCT4, SOX2, NANOG, and other pluripotency TFs still needs to be better studied.

The COP9 signalosome (CSN) is an important protein complex that prevents protein degradation, and was previously implicated in pluripotency maintenance (Chia et al., 2010). Experiments using knockdown (KD) mESCs for COPS2 protein, a specific subunit of the CSN, showed that COPS2 regulates protein stability of NANOG by its independent and direct interaction with the α-helixes 2 and 3 of NANOG’s homeobox domain, preventing its degradation by the proteasome (Zhang et al., 2016). Further, the levels of NANOG remain unaltered after the replacement of four lysine residues for arginine in its C-terminal domain and subsequent KD of COPS2, indicating this region is a strong candidate for ubiquitination, serving as a signalization for UPS degradation (Zhang et al., 2016).

More specifically, it has been previously demonstrated that OCT4 can be post-translationally modified by ubiquitin in both mESCs and hESCs. WWP2 protein was identified as the first to post-translationally modify OCT4, functioning as an E3 ligase (Xu et al., 2004). WWP2 promotes degradation of OCT4 in a dosage-dependent and also enzymatic activity-dependent manner through the 26S proteasome (Figure 1A), since OCT4 protein level progressively decreases with the increase of WWP2 expression level, and WWP2 silencing (by iRNA and shRNA) elevates OCT4 levels (Xu et al., 2009). Further, OCT4 has been shown to suffer SUMOylation at lysine residue 118 (SUMO-1 acceptor site) in mESCs, and the disruption of this modification can lead to the degradation of OCT4 by 26 proteasome and consequent impairment in self-renewal (Zhang et al., 2007).

Interestingly, evidence shows that WWP2 also plays a role in the regulation of SOX2. WWP2 HECT domain recognizes methylation on lysine 119 (K119me) of SOX2, modification that stimulates SOX2 ubiquitination and subsequent degradation via proteasome pathway (Fang et al., 2014). Treatment with MG132, a potent proteasome inhibitor, led to prominently increased levels of SOX2 K119me. The methylation in SOX2 is catalyzed by the enzyme SET7 which, intriguingly, also inhibits the transcriptional activity of SOX2 (Fang et al., 2014). On the other hand, in ESCs, direct phosphorylation of SOX2 at threonine 118 by AKT1 is a prevalent protection mechanism acting in SOX2 protein stability (Jeong et al., 2010), and inhibits methylation by SET7 at K119, stabilizing SOX2 levels, being crucial in aspects such as self-renewal and differentiation potential (Fang et al., 2014). The mechanism of AKT-mediated phosphorylation as a protector for degradation by the UPS has also been recently identified in esophageal cancer stem cells, where phosphorylation of SOX2 on threonine 116 protects the degradation mediated by the ubiquitin E3 ligase UBR5 (Wang et al., 2019). It is interesting to note that UBR5 has also been identified as an important factor in the regulation of proteostasis in hESCs and iPSCs. UBR5 is upregulated in hESCs and iPSCs derived from Huntington disease patients (Koyuncu et al., 2018). Both protein and mRNA levels are downregulated as these cells undergo differentiation (Koyuncu et al., 2018), indicating an important role of this protein in PSCs in health as well as disease. Given that PSCs and cancer stem cells share several features, the importance of UBR5 and other E3 ligases regulating the levels of TFs in PSCs should be further studied.

NANOG was previously described as a target for polyubiquitylation by the F-box protein family member FBXW8, and consequent degradation by proteasome (Kim et al., 2014). When phosphorylated by ERK1, NANOG binds to FBXW8, and experiments using mESCs and hESCs overexpressing FBXW8 resulted in a decrease in the half-life of endogenous NANOG. On the other hand, evaluation of alkaline phosphatase expression, a known pluripotency indicator, in FBXW8 KD cells, indicated decreased differentiation (Kim et al., 2014). The results suggest a key role of FBXW8, as a E3 ligase, in the regulation of NANOG levels in PSCs, by controlling its degradation.

The role of deubiquitinases (DUBs) started to be investigated in the context of proteostasis maintenance in PSCs. In brief, DUBs remove ubiquitin from protein substrates in order to maintain their targets stability. A recent study in mESCs, scanning for potential deubiquitinases regulators of NANOG, found that deubiquitinase ubiquitin-specific protease 21 (USP21) stabilizes NANOG protein levels removing K48-linked polyubiquitylation (Liu et al., 2017). USP21 directly interacts with NANOG through its ubiquitin carboxyl-terminal hydrolase domain (UCH), and the phosphorylation of USP21 by ERK1 prevents this binding, leaving this site exposed for ubiquitination, ultimately leading to NANOG degradation by the UPS (Jin et al., 2016). Another USP protein was recently reported as an indirect regulator for pluripotency TFs expression. USP26 inhibits expression of pluripotency core genes by physically binding to the members of the Protein Regulator of cytokinesis 1 (PRC1) complex, CBX4, and CBX6, preventing K48-linked polyubiquitination in these targets (Ning et al., 2017). The accumulation of CBX4 and CBX6 inhibits the expression of the SOX2 and NANOG genes, increasing the occupancy of their promoters, leading to reduction in pluripotency (Ning et al., 2017).

A summary of the proteasome-related proteins presented in this section and their function in PSCs can be found in Table 2. The evidence presented here points out the great relevance of studying the UPS in the context of PSCs, both as a possible model to better understand this system in health and diseases, and as a means of understanding the mechanisms governing the unique biology of these cells. Although many studies have been, and are still being conducted to explore these aspects, much remains to be described. It will be very interesting to follow what will be done in order to better understand the direct or indirect relationship between the great number of molecules involved in the UPS with the regulation of pluripotency TFs levels, or with other mechanisms important for PSCs maintenance, such as cell cycle control or self-renewal.

## Histone Chaperones in PSCs

As mentioned before, several factors affect the expression of pluripotency genes, and epigenetic modulations of transcription have been broadly studied to gain insight into the mechanisms which rule the rapid proliferation and turnover of pluripotent cells into differentiation. In the context of chaperone proteins, histone chaperones have recently been identified as important factors in regulating pluripotency. Here, we discuss the importance of histone chaperones in the modulation of transcription in PSCs, mainly ESCs, and their role in maintaining pluripotency through epigenetic modifications.

Chromatin in eukaryotes is organized in complexes called nucleosomes composed of 147 pairs of bases of DNA associated with a core of small basic proteins, the histones, which form an octamer of two copies of each protein H2A, H2B, H3, and H4, binding a linker histone H1 (Biterge and Schneider, 2014; Tessarz and Kouzarides, 2014). Histones can be canonical, essentially expressed during S-phase and incorporated to the nucleosome during DNA replication, or replacement histones (known as histone variants) which are incorporated into chromatin by specific histone chaperones during the cell cycle and can interact with several chromatin modifiers modulating the chromatin conformation (Biterge and Schneider, 2014). Beyond architectural functions, variant histones can regulate transcription, DNA repair and replication through covalently modifications at their flexible N- or C-terminal tails and globular domains, modulated by chromatin-modifying enzymes, which lead to a more open conformation of chromatin and allow DNA interaction with several molecules, including TFs (Strahl and Allis, 2000; Choi and Howe, 2009).

Histone chaperones are molecules that associate with histones and present an important role in histones dynamics. These chaperones are responsible for the transferring of histones to the DNA, and they can modulate histones modifications as acetylation and methylation or remodeling nucleosomes during transcription, among other important functions. Histone chaperones can work single, as the chaperone anti-silencing function 1 (ASF1), or form complexes as chromatin assembly factor 1 (CAF1) and the facilitates chromatin transcription (FACT), presenting relevant roles in post-translational histone modifications (De Koning et al., 2007; Tessarz and Kouzarides, 2014).

Embryonic stem cells present rapid changes in transcription associated with transition from pluripotency to a more differentiated state, therefore their chromatin is characterized by an open state with a less condensed structure and predominance of variant histone modified post-translationally (i.e., methylation and acetylation of H3K4) involved in transcription activation (Gaspar-Maia et al., 2011). One of the most important mechanisms by which ESCs maintain the open chromatin state is through the deposition of specific histone variants, for example H3.3, which is located at the −1 position in promoters of genes expressed in ESCs, and is commonly associated with more active transcription and decreased methylation of H3K9, a mark of condensed chromatin (heterochromatin) (Goldberg et al., 2010; Schlesinger et al., 2017). The chaperone histone regulator A (HIRA) is responsible for H3.3 deposition in pluripotent cells during replication and co-localizes with the transcriptional active form of histone H3K4 methylated (Goldberg et al., 2010), playing important roles in pluripotency and differentiation (Figure 1A). Moreover, HIRA is highly expressed in the promoters of developmentally regulated genes in ESCs and is necessary for H3.3 enrichment at genome-wide transcriptionally active and repressed genes in mESCs (Figure 1A) and neural precursor cells (Goldberg et al., 2010).

HIRA is also required in mESC for the formation of H3.3 complex with polycomb repressive complex 2 (PRC2), which control gene transcription during lineage commitment in these cells through trimethylation of lysine 27 on histone H3 (H3K27me3) (Banaszynski et al., 2013). This complex is responsible for the proper establishment of H3K27me3 at the promoters of developmentally regulated genes and in bivalent domains, characterized by the presence of the variant histones H3K4me3 (activation-associated) and H3K27me3 (repression-associated) (Bernstein et al., 2006; Banaszynski et al., 2013). HIRA can also modulate hESCs self-renewal through its interaction with prohibitin (PHB) promoting transcription of isocitrate dehydrogenase genes (IDHs), leading to the production of α-ketoglutarate, which in turn participates in metabolic processes that support pluripotency of hESCs (Zhu et al., 2017).

Runt-related transcription factor 1 (RUNX1) is essential for hematopoietic cells transition and HIRA can modulate RUNX1 targets participating of mESCs differentiation process to hemogenic endothelium through its interaction with RUNX1 and deposition of H3.3 variant (Scambler et al., 2015). Additionally, H3.3 deposition by HIRA during early embryogenesis is required for developmental reprogramming, since the loss of HIRA in female mice impairs the deposition of paternal core histone and compromise the reactivation of maternal genome (Lin et al., 2014).

Transcriptional regulator ATRX (ATRX) is a member of SNF2 family of chromatin remodeling factors and presents histone chaperone activity, forming a chromatin-remodeling complex with the death domain-associated protein DAXX. ATRX and DAXX associate with H3.3 in a HIRA-independent manner modulating H3.3 deposition at telomeres and repression of telomeric RNA in mESCs (Goldberg et al., 2010). DAXX functions as an H3.3-specific chaperone in mESCs assembling H3.3/H4 tetramers on DNA templates, and the ATRX–DAXX complex modulates the remodeling of H3.3-containing nucleosomes bounding to telomeric chromatin or pericentric repeats (Lewis et al., 2010). Interestingly, it was demonstrated that DAXX and ATRX complex are responsible for H3.3 incorporation in transposable elements containing long terminal repeats, which present regions enriched with both H3.3 and H3K9me3, those able to regulate adjacent and endogenous genes in mESCs (Elsässer et al., 2015).

Anti-silencing function 1 is the most conserved chaperone of histone 3 and 4 and is associated with nucleosome assembly, transcriptional downregulation and DNA damage response (Thuret et al., 2005). In murine, two paralogous genes *ASF1a* and *ASF1b* are present; ASF1a depletion is embryonic lethal while ASF1b was correlated with differentiation potential during early embryonic stages and gonad development (Messiaen et al., 2016). Additionally, it has been demonstrated that ASF1 binds histones H3.1 and H3.3 in mESCs (Lewis et al., 2010). In hESCs, ASF1a can affect the expression of pluripotency genes through the acetylation of H3K56, a histone state that represents the epigenetic mark of hESC (Figure 1B; Gonzalez-Muñoz et al., 2014). Interestingly, when the TFs NANOG, SOX2 and OCT4 bind to their target gene promoters it is common to observe the presence of the histone variant H3K56ac at these locations and, since ASF1 is required for the acetylation of H3K56, ASF1 depletion leads to H3K56ac decrease, and, consequently reduces expression of pluripotency markers and promotes differentiation (Tan et al., 2013). Moreover, it was demonstrated that the induced expression of both ASF1a and OCT4 in human dermal fibroblasts is able to reprogram these cells into undifferentiated iPSCs (Figure 2; Gonzalez-Muñoz et al., 2014).

Chromatin assembly factor-1 (CAF-1) complex is formed by 3 subunits P150, P60, and P48/RbAp48a and is related to processes involved in DNA synthesis and repair (De Koning et al., 2007), and also chromatin remodeling in ESCs (Houlard et al., 2006). CAF-1 is the histone chaperone that mediates H3 deposition during S-phase, associates with H3.1, transports H3.3 in the absence of DAXX in mESC and its dominant negative leads to developmental arrest before 16-cell stage (Lewis et al., 2010; Filipescu et al., 2013). The lack of one of CAF-1 subunits (P150) induces the complete loss of H3.1 and H3.2 and impairs mouse blastocysts stage development, which present modified cellular structures such as nuclear elongation and the absence of heterochromatin foci (Akiyama et al., 2011). Houlard and Filipescu, in their respective studies, demonstrated that CAF-1 associated with histone H3.2 is responsible for heterochromatin proper architecture in early development, it may contribute to gene expression during this period of development, and is also required for early gastrulation (Houlard et al., 2006; Filipescu et al., 2013). The depletion of CAF-1 subunit P150 in mESCs affects the structure of heterochromatin, which is not observed in somatic cells, leading to its decondensation and the loss of clustering and mislocalization of pericentric heterochromatin domains (Houlard et al., 2006). Moreover, CAF-1 knockdown increases H2AX phosphorylation and the developmental arrest of mouse embryos, avoiding retrotransposons appropriate silencing and damaging genome integrity, since CAF-1 is responsible for modulating the replacement of H3.3 for H3.1/3.2 and leads to the deposition of other repressive histones as H3K9me3, H3K9me2, H3K27me3, and H4K20me3 in preimplantation mouse embryos (Hatanaka et al., 2015).

Cheloufi and colleagues recently demonstrated the participation of CAF-1 in the reprogramming of mouse fibroblasts into iPSCs, since the negative modulation of CAF-1 expression produces a decrease in somatic cells heterochromatin domains, increasing the binding of SOX2 to pluripotency specific targets and somatic cellular plasticity (Cheloufi et al., 2015; Cheloufi and Hochedlinger, 2017). Interestingly, CAF-1 suppression also allows ESCs to acquire characteristics of an early developmental state, resembling totipotent 2-cell (2C) blastomeric stage of the preimplantation embryo (Yankulov, 2015).

The histone chaperone FACT is a complex composed by two subunits, SSRP1 and SPT16, and participates in transcription elongation (Mylonas and Tessarz, 2018). In mESC, FACT depletion causes a mis-activation of transcription start sites, which deregulates developmental and replication-associated genes leading to an increase in proliferation concomitant with neural commitment (Mylonas and Tessarz, 2018). FACT has been associated with OCT4 and its depletion damages mESC pluripotency and survival during early blastocyst stage (Cao et al., 2003; Gaspar-Maia et al., 2009; Pardo et al., 2010; Ding et al., 2012). Shakya et al. (2015) demonstrated that OCT4 recruits FACT complex, binds specifically SPT16 subunit of FACT in transcription sites to promote the removal of H3 histone from critical pluripotency targets such as OCT4, SOX2, and NANOG during reprograming. Moreover, it was demonstrated that the depletion of SPT16 subunit of FACT in mouse fibroblasts impair cellular reprograming and iPSCs generation (Shen et al., 2018).

Several other histone chaperones have presented important functions on ESCs biology and somatic cells reprograming. The mammalian high-mobility group AT-hook 2 (HMGA2) are highly expressed in ESCs and not translated in somatic normal cells, working as a replication fork chaperone stabilizing its integrity, in a DNA repair mechanism. During the proliferation of highly replicating cells as ESCs or cancer cells, HMGA2 expression is irrelevant, however, when forks are arrested; HMGA2 binds with high affinity to branched DNA, stabilizing stalled forks and preventing DNA mutations (Yu et al., 2014).

Another histone chaperone, nucleoplasmin-2 (NPM2), was recently associated with human dermal fibroblasts reprogramming. NPM2 associated with its histone variants TH2A and TH2 can improve the reprogramming modulated by OCT4, SOX2, KLF4, and c-MYC, generating iPSCs in a more naïve state compared to the classical TFs alone (Figure 2; Shinagawa et al., 2014; Fernández-Rivero et al., 2016). NPM2 phosphorylated form can also improve murine reprograming inducing an open chromatin structure and leading to reprogramming using only KLF4 and OCT4 (Shinagawa et al., 2014). Another NPM, the histone chaperone nucleoplasmin 3 (NPM3) is highly expressed in murine PSCs compared to differentiated cells and works as a chromatin remodeling protein, which modulates mESCs replication capacity, increasing proliferation (Motoi et al., 2008).

SPT6 is a histone chaperone and works as a transcription elongation factor in mESCs, modulating negatively H3K27 acetylation and methylation through its interaction with PRC2 core subunits, which maintains pluripotency and avoids differentiation mediated by the accumulation of H3K27me3 deposition at ESC critical super-enhancers (Robert, 2017). SET is a histone chaperone, which expression is modulated by OCT4, and is expressed during human early development and presents two isoforms, SETα and SETβ, each one controlling ESCs proliferation and differentiation, respectively (Edupuganti et al., 2017).

Finally, it is well known that PSCs present rapid turnovers to maintain an undifferentiated state or for entry into cellular commitment. Epigenetic mechanisms of gene activation that depends on histone variants allow these rapid turnovers observed in stem cells, especially PSCs, and histone chaperones are determinant for the refined work of histones modifications. Briefly, here we described how histones chaperones present relevant roles on development, pluripotency maintenance and somatic cell reprograming, as their main function is to modulate these essential epigenetic changes related to histones modifications and deposition, which have great participation on the activation and repression of TFs. Table 1 compile the main histone chaperones and their role in pluripotency and differentiation. In addition to the histone chaperones, other chaperones are closely involved to maintain the pluripotent state, and their functions in PSCs biology will be explored in the next sessions.

## PN and Heat Shock Chaperones in PSCs

Elements of the PNs are enhanced in PSCs, such as chaperones and co-chaperones. Chaperones are classified in heat-shock proteins (HSPs) such as small heat shock proteins (sHSPs), HSP60, HSP70, HSP90, and HSP100. HSP70 and HSP90 are the main families of chaperones induced in response to cellular stress and they act along TFs known as heat shock factors (HSF), which regulate several genes encoding chaperones (Hartl et al., 2011; Saibil, 2013).

The heat shock factor 1 (HSF1) creates a dormant complex with HSP90 and HSP70 under balanced conditions, however, in the presence of misfolded proteins and cytotoxic protein aggregation, chaperones detach from HSF1, which then induces members of the PN to decrease production of new clients (Saibil, 2013). When the proteotoxic stress is reduced, the HSF1 complex reassembles and the system returns to balance. Likewise, other stress-inducible chaperones are expressed in the cytosol and within organelles to create arrangements that keep non-native proteins in solution and control *de novo* folding to establish functional structures (Saibil, 2013). HSPs are abundantly expressed in PSCs than in terminally differentiated cells, providing enhanced stress response competence for PSCs which is essential for the maintenance of stemness (Saretzki, 2004).

Both protein quality control and the maintenance of the PN are indispensable for cellular biology and for the health of whole organism. Important additional pathways such as the cytosolic stress response and the unfolded protein response (UPR) are part of this complex that contributes to circumvent the accumulation of misfolded molecules (Arndt et al., 2010). However, the PN is prone to failure, despite its regulatory mechanisms, and it opens a gap for pathological protein aggregate deposits. It is considered that aggregate formation confers on the disease protein a toxic gain of function (Dimant et al., 2012). Deficiencies in proteostasis related to aggregation have been shown to facilitate the development of several illnesses, including neurodegeneration, diabetes, cystic fibrosis, and cancer (Kakkar et al., 2014). The importance of specific families of chaperones and co-chaperones in the context of pluripotency will be addressed in subsequent sessions.

### HSP90-HOP-HSP70 Complex

Heat shock protein 90 (HSP90) is a highly conserved stress response protein in eukaryotic cells, being the most abundant protein in unstressed cells, modulates the activity, turnover and trafficking of several proteins and participates of signal transduction. HSP90 forms heterocomplexes with different proteins through the formation of a complex composed by HSP90 and another four chaperones – HSP70, HSP Organizing Protein (HOP), HSP40, and p23 – binding to several clients as steroid receptors. The assembly requires HSP90 and HSP70 bound by HOP and incorporates HSP40 and p23 during the process, assisting the folding and refolding of naïve proteins for properly active conformation (Pratt and Toft, 2003). This chaperone machinery is ATP-dependent, in which HSP90 binds to ATP and p23 stabilizes the complex. Both HSP70 and HSP40 also interact after ATP binding and HOP brings together both complexes preparing the chaperone machinery to receive a substrate, that can be a receptor, a protein kinase or a TF, among other proteins associated with signaling transduction (Pratt and Toft, 2003). The HSF are TFs responsible for increasing heat shock proteins expression during stress conditions and, on the other hand, HSP90 complex is responsible for sequester HSFs in normal conditions avoiding stress response activation in a feedback loop (Nichols et al., 1998).

Several studies support the importance of HSP90-HOP-HSP70 complex on PSCs biology. Recent data from our group determined that HOP, HSP70, and HSP90 are found as cargo of extracellular vesicles (implicated in intercellular communication) of mESCs, suggesting their participation in processes related to development, since the microenvironment is important for pluripotency maintenance through the modulation of several signaling pathways (Cruz et al., 2018a,b).

Constitutive HSP90 is required for mouse embryos development and maintenance of pluripotency via HOP-STAT3 interaction (Figure 1A); moreover, ESCs express a specific conjunct of types of chaperones related to this complex as HSP70 protein 4 (HSPA4) and HSP90β (HSPCB). In addition, stem cells including ESCs present an increase in the expression of HSP70 protein 5 (HSPA5), HSP70 protein 8 (HSPA8), and HOP (Fan, 2012). HSP70 protein 8 (HSPA8) is highly expressed on the surface of hESCs (Figure 1B) and co-expressed with specific stem cells markers, for example stage-specific embryonic antigen 3 and 4 (SSEA3/4) (Son et al., 2005).

HSP90 has been described in literature as an essential molecule for pluripotency maintenance. The inhibition of HSP90 expression decreases OCT4, NANOG and pSTAT3 levels and leads to mESCs differentiation, preferentially to the mesoderm layer. HSP90 associates with OCT4 and NANOG to protect them from ubiquitin proteasome degradation (Figure 1A) and is also able to modulate OCT4 mRNA levels supporting pluripotency maintenance (Bradley et al., 2012). During heat shock in hESCs, there is a hyperactivation of the MAP kinases and hESCs starts to differentiate through the increase of the HSF1 transcription factor, HSP90 releases HSF1 to answer the stress stimuli and HSF1 in turn binds to OCT4 promoter region leading to its repression (Byun et al., 2013).

Setati et al. (2010) described that HSP90 is responsible for STAT3 translocation to the nucleus during the activation of the JAK-STAT3 pathway, which occurs through LIF stimuli during mESCs self-renewal (Figure 1A). STAT3 translocation to the nucleus by HSP90 leads to STAT3 association with the NANOG promoter region modulating pluripotency of mESCs (Okumura et al., 2011). Negative modulation of NANOG transcription occurs through constitutive HSP90 sequester by TRIM8, which impairs its association with STAT3 and, consequently translocation to the nucleus, avoiding pluripotency signaling via LIF pathway (Setati et al., 2010). One of HSP90 clients is the transcription factor HNF4A, their binding in iPSCs is able to modulate specifically differentiation from these to endoderm-derived hepatic progenitor cells (Jing et al., 2017).

HSP90 partner HOP is essential for mouse development since its knockout (KO) is embryonic lethal and HOP KO mouse do not reach E10.5 development stage (Beraldo et al., 2013). HOP was suggested to modulate STAT3 phosphorylation and, through its binding to HSP90, participate in STAT3 translocation to the nucleus, being another important chaperone in mESC pluripotency by LIF/JAK/STAT3 signaling pathway (Figure 1A; Longshaw et al., 2009). Longshaw et al. (2009) demonstrated STAT3 accumulation into the cytoplasm, decreased phosphorylation and STAT3 mRNA levels after HOP depletion in mESC. Moreover, HOP knockdown mESCs presented a decrease in NANOG mRNA levels and decreased pluripotency observed in the formation of embryoid bodies (EBs).

HSP70 is constitutively expressed and its expression can be inducted during stress. In hESCs, constitutive HSP70 expression is slightly increased compared to somatic cells and is located into the cytoplasm and nucleus independent of differentiation status. However, in hESCs HSP70 can also be found on cell surface (Figure 1B), which is not observed in somatic cells, suggesting HSP70 is a possible surface marker for pluripotent cells (Alekseenko et al., 2012).

The HSPS from the family HSP70 HSPA1A (HSP70A1), HSPA1B (HSP70B1), and HSPA9A (HSP70A9, mortalin) are highly expressed in mESCs compared to differentiated cells and are associated with increased resistance of these cells against genotoxic stress (Saretzki, 2004). Additionally, it was demonstrated that during differentiation of some mESC lines there is a decrease in expression of both HSP70 and its partner HOP (Baharvand et al., 2008).

Recently, it was demonstrated that upregulation of HSP70 in iPSCs caused by stress as hypoxia promotes survival through the inhibition of apoptosis pathways, however, it also inhibits STAT3 phosphorylation leading to differentiation (Figure 2) and increased resistance to stress, since iPSCs are highly sensitive to conditions adverse to homeostasis (Brodarac et al., 2015). HSP70 participates in the early differentiation modulated by epigenetic factor histone deacetylase (HDAC) of mESCs and hESCs, through the activation of JNK and PI3K/AKT pathways (Park et al., 2011). In mESC, HSP70 expression is enriched by the MAPK signaling pathway though JNK, ERK, and p38 modulating several responses to stress related to cell survival and anti-apoptosis processes (Nishitai and Matsuoka, 2008).

Differentiation of mESCs into neurogenic EBs (NEBs) leads to a decrease in the expression of mortalin, a chaperone from the family of HSP70 (HSP70A9) (Battersby et al., 2007). Another protein from HSP70 family, HSPA1b, also presents a significant decrease in its expression during differentiation and its remarkable that this downregulation occurs and is detectable before the expression of differentiation markers, supporting a role for HSP70 proteins in modulating differentiation (Saretzki et al., 2007).

### HSP60, HSP40, and Small HSPs Families

HSP60, also known as HSPD1, is a mitochondrial chaperonin, involved in the synthesis and transportation of mitochondrial proteins from the cytoplasm to the mitochondria (Cappello et al., 2008). HSP60 is able to interact with different HSPs, such as HSP10, forming a complex that mediates protein folding (Okamoto et al., 2017), and with mitochondrial HSP70 (HSP70A), also known as mortalin, that have a role in cell proliferation and stress (Wadhwa et al., 2006). Studies shows that HSP60 have an important role in the biology of pluripotent cells. HSP60 deficiency in progenitor cells from the intestinal crypt compartment induces mitochondrial dysfunction, which leads to a decrease in stemness and cell proliferation (Berger et al., 2016). In mESCs, HSP60 expression decreases with cell differentiation, and its depletion caused a decrease in OCT4 expression, inhibiting proliferation and self-renewal, and increasing apoptosis in a caspase-3 independent-manner (Seo et al., 2018). Besides that, HSP60 knockdown also decreased EBs size and increased S-phase cells in mESCs (Seo et al., 2018). As seen in mouse cells, rabbit ESCs (rESCs) HSP60 levels are also increased when compared to differentiated cells, suggesting that proteins from the HSP family might have an important role in maintaining the undifferentiated status of embryonic cells (Intawicha et al., 2013). Interestingly, studies in cancer showed that *HSP60* is a target gene of c-MYC (Tsai et al., 2008), but is also able to induce c-MYC expression, as *HSP60* overexpression increased the proteins levels of c-MYC (Yan F.Q. et al., 2015).

HSP40 can interact with HSP70 and modulate its ATPase activity through stabilizing its interaction with subtracts (Prinsloo et al., 2009). HSP47, also known as SERPINH1, is required for the proper assembly of triple-helical procollagen molecules, and is highly expressed during mESC differentiation into smooth muscle cells (SMC) (Wong et al., 2014). Depletion of HSP47 leads to a decrease in the differentiation of mESC to mSMC and, in the same way, an overexpression of HSP47 leads to an increase in differentiation (Wong et al., 2014). HSP47 was identified as a marker for endodermal differentiation in mouse teratoma (Wang and Gudas, 1988). In bovine embryos, all HSP40 family members are upregulated during degeneration in an anti-apoptotic system, while normal blastocysts highly express HSP70 family members as HSPA5 and HSPA8 (Zhang et al., 2011). Both HSP40 and HSP47 are involved in the development of mouse limbs, along with other chaperones including from HSP70 family and small HSPs (Zhu et al., 2010; Yan F.Q. et al., 2015). A study using Glioblastoma Multiforme stem cells showed that HSP47 is capable of modulating TGF-β, inducing cell survivability, self-renewal, and increasing the number of stem cell-like cells within the tumor (Jing et al., 2017). Still, more studies elucidating the possible roles of the HSP40 family in the maintenance of stem cells are necessary.

The small HSP family is composed out of HSP of low molecular weight. Small HSPs present relevant roles in mouse development, for example HSP10, HSP22, and HSP25 that are involved in murine limb development (Zhu et al., 2010; Yan Z. et al., 2015). In adipose tissue-derived stem cells, HSP32, also known as hemeoxygenase-1, has its expression increased after the cells are left in 43°C for 1 h, which in turn increases the prevalence of live cells after a frozen-thawn cycle, reducing oxidative stress damage (Shaik et al., 2017). Besides that, a study in Chronic Myeloid Leukemia, a hematopoietic stem cell disease, shows that HSP32 is capable of increasing the survivability of those cells, through protection against apoptosis (Mayerhofer et al., 2008). Another small HSP with an interesting role in stemness is HSP27, also known as heat shock protein beta-1 (HSPB1). A study shows a downregulation of HSP27 after mESC differentiate into NEB (Prinsloo et al., 2009). Besides that, HSP27 is capable of regulating STAT3 expression, with their expression levels correlating directly in prostate cancer cells (Rocchi et al., 2005). HSP27 is also able to regulate EIF4E and eIF4G levels in the first trimester human placentae, indicating that HSP27 could alter placental protein translation (Shochet et al., 2016). HSP27 also seems to have an important role in neuronal differentiation, with its overexpression leading stem cells to arrest neuronal differentiation, and its expression decreasing as embryonic neuronal development occurred *in vivo* (Cheng et al., 2016). In placenta-derived multipotent cells (PDMCs) HSP27 inhibition leads to NANOG cleavage mediated by caspase-3 activation (Cheng et al., 2016).

In zebrafish development both HSP27 family members, HSPB7 and HSPB12, are modulated by GATA4 protein and modulate cardiac development, since their depletion leads to a disruption in normal cardiac morphogenesis perturbing Kuppfer’s vesicle morphology (Rosenfeld et al., 2013). Indeed, HSPB7 is highly expressed in cardiac cells and its depletion in mouse lacks the normal development of the heart, leading to embryo lethality before embryonic day 12.5 (Wu et al., 2017). HSPB1 (HSP27 family) also presents an important role in zebrafish development modulating the growth of myofibrils of skeletal muscle (Middleton and Shelden, 2013). In avian blastoderms, the expression of the sHSP HSP25 is required during the embryo development for the expression of pluripotency genes and for resistance against apoptosis (Hwang et al., 2016).

In light of all these findings, it is remarkable that the participation of heat shock proteins in the biology of PSCs. HSPs can modulate several signaling cascades including pluripotency essential pathways. They also modulate both expression and localization of TFs and relevant proteins, and their expression is essential for the development of the embryos of many species. Together, these studies demonstrate the importance of stress response and HSPs function in development and pluripotency maintenance of PSCs.

## Perspectives

A better overview of the nature of pluripotency holds great promise for therapeutic approaches. Unveiling the exact molecular mechanisms that govern the ability of ESCs and iPSCs to differentiate in all cell types to give rise to an adult organism is essential to exploit these cells both in regenerative medicine and to model human diseases pathogenesis including cancer and neurodegenerative disorders.

A regulatory core (NANOG, OCT4, and SOX2) and other TFs, epigenetic regulators and specific signaling pathways are key elements to orchestrate pluripotency. Although these well-established TFs are of undeniable importance for pluripotency maintenance, the search for additional TFs that are able to interact with core factors to regulate processes related to pluripotency should be enhanced, and the role of an expanded core has just begun to be dissected. Moving forward to the debate on the importance of TFs for pluripotency and stemness regulation, this will be a fruitful area for further research.

As presented here, emerging evidence points out the network plasticity of chaperones controlling the activities of key players involved in pluripotency maintenance. Chaperones play roles from shaping chromatin dynamic to controlling transcriptional regulation of pluripotency genes, to the assistance of proper folding of these genes when translated into proteins in different PSCs models (Figures 1, 2). The broad spectrum of chaperones activities in the essential processes of stemness control reveals that stem cells exhibit intrinsic proteostasis mechanism, which can be included as a component of the pluripotency regulatory network. In somatic cells a collapse in proteostasis underlies several diseases including neurodegenerative disease and cancer, conversely, PSCs exhibit the remarkable capacity to correct and repress proteome imbalance, indicating that additional investigation is required for an in-depth understanding of enhanced protein homeostasis in stem cell biology.

This review brings together classical and recent research on the control of pluripotency, going through broad important cellular processes related to this unique and promising feature. The diversity of these processes embraces different levels of cellular regulation and shows how complex is the understanding of the pluripotency context.

## Author Contributions

CF and RI conceived the presented idea and proof outline, did the literature review, organized the tables, and wrote distinct topics. MP did the literature review and wrote a topic. MM-E did the literature review, wrote a topic, and prepared the illustration (art-Figures 1, 2). ML conceived the presented idea and proof outline, wrote topics, and reviewed the manuscript.

## Conflict of Interest Statement

The authors declare that the research was conducted in the absence of any commercial or financial relationships that could be construed as a potential conflict of interest.

## References

[B1] Akiyama T. Suzuki O. Matsuda J. Aoki F. (2011). Dynamic replacement of histone H3 variants reprograms epigenetic marks in early mouse embryos. *PLoS Genet.* 7:e1002279. 10.1371/journal.pgen.1002279 21998593 PMC3188537

[B2] Alekseenko L. L. Zemelko V. I. Zenin V. V. Pugovkina N. A. Kozhukharova I. V. Kovaleva Z. V. (2012). Heat shock induces apoptosis in human embryonic stem cells but a premature senescence phenotype in their differentiated progeny. *Cell Cycle* 11 3260–3269. 10.4161/cc.21595 22895173 PMC3466525

[B3] Arndt V. Dick N. Tawo R. Dreiseidler M. Wenzel D. Hesse M. (2010). Chaperone-assisted selective autophagy is essential for muscle maintenance. *Curr. Biol.* 20 143–148. 10.1016/j.cub.2009.11.022 20060297

[B4] Baharvand H. Fathi A. Gourabi H. Mollamohammadi S. Salekdeh G. H. (2008). Identification of mouse embryonic stem cell-associated proteins. *J. Proteome Res.* 7 412–423. 10.1021/pr700560t 18047272

[B5] Banaszynski L. A. Wen D. Dewell S. Whitcomb S. J. Lin M. Diaz N. (2013). Hira-dependent histone H3.3 deposition facilitates prc2 recruitment at developmental loci in ES cells. *Cell* 155 107–120. 10.1016/j.cell.2013.08.061 24074864 PMC3838450

[B6] Battersby A. Jones R. D. Lilley K. S. McFarlane R. J. Braig H. R. Allen N. D. (2007). Comparative proteomic analysis reveals differential expression of Hsp25 following the directed differentiation of mouse embryonic stem cells. *Biochim. Biophys. Acta Mol. Cell Res.* 1773 147–156. 10.1016/j.bbamcr.2006.08.030 17030443

[B7] Beraldo F. H. Soares I. N. Goncalves D. F. Fan J. Thomas A. A. Santos T. G. (2013). Stress-inducible phosphoprotein 1 has unique cochaperone activity during development and regulates cellular response to ischemia via the prion protein. *FASEB J.* 27 3594–3607. 10.1096/fj.13-232280 23729591

[B8] Berger E. Rath E. Yuan D. Waldschmitt N. Khaloian S. Allgäuer M. (2016). Mitochondrial function controls intestinal epithelial stemness and proliferation. *Nat. Commun.* 7:13171. 10.1038/ncomms13171 27786175 PMC5080445

[B9] Bernstein B. E. Mikkelsen T. S. Xie X. Kamal M. Huebert D. J. Cuff J. (2006). A Bivalent Chromatin Structure Marks Key Developmental Genes in Embryonic Stem Cells. *Cell* 125 315–326. 10.1016/j.cell.2006.02.041 16630819

[B10] Bharathan S. P. Manian K. V. Aalam S. M. M. Palani D. Deshpande P. A. Pratheesh M. D. (2017). Systematic evaluation of markers used for the identification of human induced pluripotent stem cells. *Biol. Open* 6 100–108. 10.1242/bio.022111 28089995 PMC5278432

[B11] Biterge B. Schneider R. (2014). Histone variants: key players of chromatin. *Cell Tissue Res.* 356 457–466. 10.1007/s00441-014-1862-4 24781148

[B12] Boyer L. A. Tong I. L. Cole M. F. Johnstone S. E. Levine S. S. Zucker J. P. (2005). Core transcriptional regulatory circuitry in human embryonic stem cells. *Cell* 122 947–956. 10.1016/j.cell.2005.08.020 16153702 PMC3006442

[B13] Bradley E. Bieberich E. Mivechi N. F. Tangpisuthipongsa D. Wang G. (2012). Regulation of embryonic stem cell pluripotency by heat shock protein 90. *Stem Cells* 30 1624–1633. 10.1002/stem.1143 22696450 PMC3665290

[B14] Brodarac A. Saric T. Oberwallner B. Mahmoodzadeh S. Neef K. Albrecht J. (2015). Susceptibility of murine induced pluripotent stem cell-derived cardiomyocytes to hypoxia and nutrient deprivation. *Stem Cell Res. Ther.* 6 1–19. 10.1186/s13287-015-0057-6 25900017 PMC4445302

[B15] Buckley S. M. Aranda-Orgilles B. Strikoudis A. Apostolou E. Loizou E. Moran-Crusio K. (2012). Regulation of pluripotency and cellular reprogramming by the ubiquitin-proteasome system. *Cell Stem Cell* 11 783–798. 10.1016/j.stem.2012.09.011 23103054 PMC3549668

[B16] Byun K. Kim T. K. Oh J. Bayarsaikhan E. Kim D. Lee M. Y. (2013). Heat shock instructs hESCs to exit from the self-renewal program through negative regulation of OCT4 by SAPK/JNK and HSF1 pathway. *Stem Cell Res.* 11 1323–1334. 10.1016/j.scr.2013.08.014 24090933

[B17] Cao S. Bendall H. Hicks G. G. Nashabi A. Sakano H. Shinkai Y. (2003). The high-mobility-group box protein SSRP1/T160 is essential for cell viability in day 3.5 mouse embryos. *Mol. Cell. Biol.* 23 5301–5307. 10.1128/mcb.23.15.5301-5307.2003 12861016 PMC165710

[B18] Cappello F. Conway De Macario E. Marasà L. Zummo G. Macario A. J. L. (2008). Hsp60 expression, new locations, functions and perspectives for cancer diagnosis and therapy. *Cancer Biol. Ther.* 7 801–809. 10.4161/cbt.7.6.6281 18497565

[B19] Cheloufi S. Elling U. Hopfgartner B. Jung Y. L. Murn J. Ninova M. (2015). The histone chaperone CAF-1 safeguards somatic cell identity. *Nature* 528 218–224. 10.1038/nature15749 26659182 PMC4866648

[B20] Cheloufi S. Hochedlinger K. (2017). Emerging roles of the histone chaperone CAF-1 in cellular plasticity. *Curr. Opin. Genet. Dev.* 46 83–94. 10.1016/j.gde.2017.06.004 28692904 PMC5813839

[B21] Cheng Y. C. Huang C. J. Lee Y. J. Tien L. T. Ku W. C. Chien R. (2016). Knocking down of heat-shock protein 27 directs differentiation of functional glutamatergic neurons from placenta-derived multipotent cells. *Sci. Rep.* 6 1–15. 10.1038/srep30314 27444754 PMC4957209

[B22] Chia N. Y. Chan Y. S. Feng B. Lu X. Orlov Y. L. Moreau D. (2010). A genome-wide RNAi screen reveals determinants of human embryonic stem cell identity. *Nature* 468 316–320. 10.1038/nature09531 20953172

[B23] Choi J. K. Howe L. J. (2009). Histone acetylation: truth or consequences? *Biochem. Cell Bio* 87 139–150. 19234530 10.1139/O08-112

[B24] Collier A. J. Panula S. P. Schell J. P. Chovanec P. Plaza Reyes A. Petropoulos S. (2017). Comprehensive cell surface protein profiling identifies specific markers of human naive and primed pluripotent states. *Cell Stem Cell* 20 874.e7–890.e7. 10.1016/j.stem.2017.02.014 28343983 PMC5459756

[B25] Cruz L. Arevalo Romero J. A. Brandão Prado M. Santos T. G. Hohmuth Lopes M. (2018a). Evidence of extracellular vesicles biogenesis and release in mouse embryonic stem cells. *Stem Cell Rev. Reports* 14 262–276. 10.1007/s12015-017-9776-7 29032399

[B26] Cruz L. Romero A. A. J. Iglesia P. R. Lopes H. M. (2018b). Extracellular vesicles: decoding a new language for cellular communication in early embryonic development. *Front. Cell Dev. Biol* 6:94. 10.3389/fcell.2018.00094 30211159 PMC6121069

[B27] De Koning L. Corpet A. Haber J. E. Almouzni G. (2007). Erratum: histone chaperones: an escort network regulating histone traffic (nature structural and molecular biology (2007) 14, (997-1007)). *Nat. Struct. Mol. Biol.* 14 997–1007. 10.1038/nsmb1318 17984962

[B28] Dimant H. Ebrahimi-Fakhari D. McLean P. J. (2012). Molecular chaperones and co-chaperones in parkinson disease. *Neuroscientist* 18 589–601. 10.1177/1073858412441372 22829394 PMC3904222

[B29] Ding J. Xu H. Faiola F. Ma’Ayan A. Wang J. (2012). Oct4 links multiple epigenetic pathways to the pluripotency network. *Cell Res.* 22 155–167. 10.1038/cr.2011.179 22083510 PMC3252465

[B30] Edupuganti R. R. Harikumar A. Aaronson Y. Biran A. Sailaja B. S. Nissim-Rafinia M. (2017). Alternative SET/TAFI promoters regulate embryonic stem cell differentiation. *Stem Cell Rep.* 9 1291–1303. 10.1016/j.stemcr.2017.08.021 28966118 PMC5639460

[B31] Elsässer S. J. Noh K.-M. Diaz N. Allis D. Banaszynski L. A. (2015). Histone H3.3 is required for endogenous retrovira element silencing in embryonic stem cells. *Science* 522 240–244. 10.1038/nature14345 25938714 PMC4509593

[B32] Emani M. R. Närvä E. Stubb A. Chakroborty D. Viitala M. Rokka A. (2015). The L1TD1 protein interactome reveals the importance of post-transcriptional regulation in human pluripotency. *Stem Cell Rep.* 4 519–528. 10.1016/j.stemcr.2015.01.014 25702638 PMC4376047

[B33] Evans M. J. Kaufman M. H. (1981). Establishment in culture of pluripotential cells from mouse embryos. *Nature* 292 154–156. 10.1038/292154a0 7242681

[B34] Fan G.-C. (2012). Genetics of stem cells. *Part A Prog. Mol. Biol. Transl. Sci.* 111 305–322. 10.1016/B978-0-12-398459-3.00014-9 22917237 PMC4422174

[B35] Fang L. Zhang L. Wei W. Jin X. Wang P. Tong Y. (2014). A methylation-phosphorylation switch determines Sox2 stability and function in ESC maintenance or differentiation. *Mol. Cell* 55 537–551. 10.1016/j.molcel.2014.06.018 25042802

[B36] Fernández-Rivero N. Franco A. Velázquez-Campoy A. Alonso E. Muga A. Prado A. (2016). A quantitative characterization of nucleoplasmin/histone complexes reveals chaperone versatility. *Sci. Rep.* 6 1–14. 10.1038/srep32114 27558753 PMC4997359

[B37] Filipescu D. Szenker E. Almouzni G. (2013). Developmental roles of histone H3 variants and their chaperones. *Trends Genet.* 29 630–640. 10.1016/j.tig.2013.06.002 23830582

[B38] Gaspar-Maia A. Alajem A. Meshorer E. Ramalho-Santos M. (2011). Open chromatin in pluripotency and reprogramming. *Nat. Rev. Mol. Cell Biol.* 12 36–47. 10.1038/nrm3036 21179060 PMC3891572

[B39] Gaspar-Maia A. Alajem A. Polesso F. Sridharan R. Mason M. J. Heidersbach A. (2009). Chd1 regulates open chromatin and pluripotency of embryonic stem cells. *Nature* 460 863–868. 10.1038/nature08212 19587682 PMC3891576

[B40] Goldberg A. D. Banaszynski L. A. Noh K. M. Lewis P. W. Elsaesser S. J. Stadler S. (2010). Distinct factors control histone variant H3.3 localization at specific genomic regions. *Cell* 140 678–691. 10.1016/j.cell.2010.01.003 20211137 PMC2885838

[B41] Gonzalez-Muñoz E. Yohanna A.-E. Hasan H. O. Jose B. C. (2014). Histone chaperone ASF1A is required for maintenance of pluripotency and cellular reprogramming. *Science* 345 822–825. 10.1126/science.1254745 25035411

[B42] Hackett J. A. Azim Surani M. (2014). Regulatory principles of pluripotency: from the ground state up. *Cell Stem Cell* 15 416–430. 10.1016/j.stem.2014.09.015 25280218

[B43] Hartl F. U. Bracher A. Hayer-Hartl M. (2011). Molecular chaperones in protein folding and proteostasis. *Nature* 475 324–332. 10.1038/nature10317 21776078

[B44] Hatanaka Y. Inoue K. Oikawa M. Kamimura S. Ogonuki N. Kodama E. N. (2015). Histone chaperone CAF-1 mediates repressive histone modifications to protect preimplantation mouse embryos from endogenous retrotransposons. *Proc. Natl. Acad. Sci.* 112 14641–14646. 10.1073/pnas.1512775112 26546670 PMC4664303

[B45] Houlard M. Berlivet S. Probst A. V. Quivy J. P. Héry P. Almouzni G. (2006). CAF-1 is essential for heterochromatin organization in pluripotent embryonic cells. *PLoS Genet.* 2:e18. 10.1371/journal.pgen.0020181 17083276 PMC1630711

[B46] Huang D. Wang L. Duan J. Huang C. Tian X. Zhang M. (2017). LIF-activated Jak signaling determines esrrb expression during late-stage reprogramming. *Biol. Open* 7:bio029264. 10.1242/bio.029264 29212799 PMC5829498

[B47] Hwang Y. S. Ko M. H. Kim Y. M. Park Y. H. Ono T. Han J. Y. (2016). The avian-specific small heat shock protein HSP25 is a constitutive protector against environmental stresses during blastoderm dormancy. *Sci. Rep.* 6 1–12. 10.1038/srep36704 27827412 PMC5101479

[B48] Intawicha P. Wang S. H. Hsieh Y. C. Lo N. W. Lee K. H. Huang S. Y. (2013). Proteomic profiling of rabbit embryonic stem cells derived from parthenotes and fertilized embryos. *PLoS One* 8:e67772. 10.1371/journal.pone.0067772 23861804 PMC3701598

[B49] Jang J. Wang Y. Kim H. S. Lalli M. A. Kosik K. S. (2014). Nrf2, a regulator of the proteasome, controls self-renewal and pluripotency in human embryonic stem cells. *Stem Cells* 32 2616–2625. 10.1002/stem.1764 24895273 PMC4165656

[B50] Jeong C. H. Cho Y. Y. Kim M. O. Kim S. H. Cho E. J. Lee S. Y. (2010). Phosphorylation of Sox2 cooperates in reprogramming to pluripotent stem cells. *Stem Cells* 28 2141–2150. 10.1002/stem.540 20945330

[B51] Jin J. Liu J. Chen C. Liu Z. Jiang C. Chu H. (2016). The deubiquitinase USP21 maintains the stemness of mouse embryonic stem cells via stabilization of Nanog. *Nat. Commun.* 7 1–15. 10.1038/ncomms13594 27886188 PMC5133637

[B52] Jing R. Duncan C. B. Duncan S. A. (2017). A small-molecule screen reveals that HSP90β promotes the conversion of induced pluripotent stem cell-derived endoderm to a hepatic fate and regulates HNF4A turnover. *Development* 144 1764–1774. 10.1242/dev.146845 28360131 PMC5450838

[B53] Kakkar V. Meister-Broekema M. Minoia M. Carra S. Kampinga H. H. (2014). Barcoding heat shock proteins to human diseases: looking beyond the heat shock response. *Dis. Model. Mech.* 7 421–434. 10.1242/dmm.014563 24719117 PMC3974453

[B54] Kim J. Chu J. Shen X. Wang J. Orkin S. H. (2008). An extended transcriptional network for pluripotency of embryonic stem cells. *Cell* 132 1049–1061. 10.1016/j.cell.2008.02.039 18358816 PMC3837340

[B55] Kim S. H. Kim M. O. Cho Y. Y. Yao K. Kim D. J. Jeong C. H. (2014). ERK1 phosphorylates Nanog to regulate protein stability and stem cell self-renewal. *Stem Cell Res.* 13 1–11. 10.1016/j.scr.2014.04.001 24793005

[B56] Koyuncu S. Saez I. Lee H. J. Gutierrez-Garcia R. Pokrzywa W. Fatima A. (2018). The ubiquitin ligase UBR5 suppresses proteostasis collapse in pluripotent stem cells from Huntington’s disease patients. *Nat. Commun.* 9 1–22. 10.1038/s41467-018-05320-3 30038412 PMC6056416

[B57] Lecker S. H. Goldberg A. L. Mitch W. E. (2006). Protein degradation by the ubiquitin–proteasome pathway in normal and disease states. *J. Am. Soc. Nephrol* 17 1807–1819. 10.1681/ASN.2006010083 16738015

[B58] Lewis P. W. Elsaesser S. J. Noh K.-M. Stadler S. C. Allis C. D. (2010). Daxx is an H3.3-specific histone chaperone and cooperates with ATRX in replication-independent chromatin assembly at telomeres. *Proc. Natl. Acad. Sci.* 107 14075–14080. 10.1073/pnas.1008850107 20651253 PMC2922592

[B59] Lin C. J. Koh F. M. Wong P. Conti M. Ramalho-Santos M. (2014). Hira-mediated H3.3 incorporation is required for DNA replication and ribosomal RNA transcription in the mouse zygote. *Dev. Cell* 30 268–279. 10.1016/j.devcel.2014.06.022 25087892 PMC4134436

[B60] Liu X. Yao Y. Ding H. Han C. Chen Y. Zhang Y. (2017). USP21 deubiquitylates nanog to regulate protein stability and stem cell pluripotency. *Signal Transduct. Target. Ther.* 2:16046. 10.1038/sigtrans.2016.46 29266131 PMC5661619

[B61] Longshaw V. M. Baxter M. Prewitz M. Blatch G. L. (2009). Knockdown of the co-chaperone hop promotes extranuclear accumulation of stat3 in mouse embryonic stem cells. *Eur J Cell Biol.* 88 153–166. 10.1016/j.ejcb.2008.09.003 18996616

[B62] Marks H. Kalkan T. Menafra R. Denissov S. Jones K. Hofemeister H. (2012). The transcriptional and epigenomic foundations of ground state pluripotency. *Cell* 149 590–604. 10.1016/j.cell.2012.03.026 22541430 PMC3398752

[B63] Martello G. Smith A. (2014). The nature of embryonic stem cells. *Annu. Rev. Cell Dev. Biol.* 30 647–675. 10.1146/annurev-cellbio-100913-013116 25288119

[B64] Martin G. R. (1981). Isolation of a pluripotent cell line from early mouse embryos cultured in medium conditioned by teratocarcinoma stem cells. *Proc. Natl. Acad. Sci. U.S.A.* 78 7634–7638. 10.1073/pnas.78.12.7634 6950406 PMC349323

[B65] Mayerhofer M. Gleixner K. V. Mayerhofer J. Hoermann G. Jaeger E. Aichberger K. J. (2008). Targeting of heat shock protein 32 (Hsp32)/heme oxygenase-1 (HO-1) in leukemic cells in chronic myeloid leukemia: a novel approach to overcome resistance against imatinib. *Blood* 111 2200–2210. 10.1182/blood-2006-11-055723 18024796

[B66] Messiaen S. Guiard J. Aigueperse C. Fliniaux I. Tourpin S. Barroca V. (2016). Loss of the histone chaperone ASF1B reduces female reproductive capacity in mice. *Reproduction* 151 477–489. 10.1530/REP-15-0327 26850882

[B67] Messmer T. von Meyenn F. Savino A. Santos F. Mohammed H. Lun A. T. L. (2019). Transcriptional heterogeneity in naive and primed human pluripotent stem cells at single-cell resolution. *Cell Rep.* 26 815.e4–824.e4. 10.1016/j.celrep.2018.12.099 30673604 PMC6344340

[B68] Middleton R. C. Shelden E. A. (2013). Small heat shock protein HSPB1 regulates growth of embryonic zebrafish craniofacial muscles. *Exp. Cell Res.* 319 860–874. 10.1016/j.yexcr.2013.01.002 23313812

[B69] Motoi N. Suzuki K. I. Hirota R. Johnson P. Oofusa K. Kikuchi Y. (2008). Identification and characterization of nucleoplasmin 3 as a histone-binding protein in embryonic stem cells. *Dev. Growth Differ.* 50 307–320. 10.1111/j.1440-169X.2008.01034.x 18462200

[B70] Mylonas C. Tessarz P. (2018). Transcriptional repression by FACT is linked to regulation of chromatin accessibility at the promoter of ES cells. *Life Sci. Alliance* 1 e201800085. 10.26508/lsa.201800085 30456357 PMC6238418

[B71] Nichols J. Smith A. (2009). Naive and primed pluripotent states. *Cell Stem Cell* 4 487–492. 10.1016/j.stem.2009.05.015 19497275

[B72] Nichols J. Zevnik B. Anastassiadis K. Niwa H. Klewe-Nebenius D. Chambers I. (1998). Formation of pluripotent stem cells in the mammalian embryo depends on the POU transcription factor Oct4. *Cell* 95 379–391. 10.1016/S0092-8674(00)81769-9 9814708

[B73] Nicola N. A. Babon J. J. (2015). Leukemia inhibitory factor (LIF). *Cytokine Growth Factor Rev.* 26 533–544. 10.1016/j.cytogfr.2015.07.001 26187859 PMC4581962

[B74] Ning B. Zhao W. Qian C. Liu P. Li Q. Li W. (2017). USP26 functions as a negative regulator of cellular reprogramming by stabilising PRC1 complex components. *Nat. Commun.* 8 1–10. 10.1038/s41467-017-00301-4 28839133 PMC5571198

[B75] Nishitai G. Matsuoka M. (2008). Differential regulation of HSP70 expression by the JNK kinases SEK1 and MKK7 in mouse embryonic stem cells treated with cadmium. *J. Cell. Biochem.* 104 1771–1780. 10.1002/jcb.21743 18348256

[B76] Okamoto T. Yamamoto H. Kudo I. Matsumoto K. Odaka M. Grave E. (2017). HSP60 possesses a GTPase activity and mediates protein folding with HSP10. *Sci. Rep.* 7:16931. 10.1038/s41598-017-17167-7 29208924 PMC5717063

[B77] Okita Y. Nakayama K. I. (2012). UPS delivers pluripotency. *Cell Stem Cell* 11 728–730. 10.1016/j.stem.2012.11.009 23217415

[B78] Okumura F. Okumura A. J. Matsumoto M. Nakayama K. I. Hatakeyama S. (2011). TRIM8 regulates Nanog via Hsp90β-mediated nuclear translocation of STAT3 in embryonic stem cells. *Biochim. Biophys. Acta Mol. Cell Res.* 1813 1784–1792. 10.1016/j.bbamcr.2011.05.013 21689689

[B79] Pardo M. Lang B. Yu L. Prosser H. Bradley A. Babu M. M. (2010). An expanded oct4 interaction network: implications for stem cell biology. *Devel. Dis. Cell Stem Cell* 6 382–395. 10.1016/j.stem.2010.03.004 20362542 PMC2860244

[B80] Park J. A. Kim Y. E. Seok H. J. Park W. Y. Kwon H. J. Lee Y. (2011). Differentiation and upregulation of heat shock protein 70 induced by a subset of histone deacetylase inhibitors in mouse and human embryonic stem cells. *BMB Rep.* 44 176–181. 10.5483/BMBRep.2011.44.3.176 21429295

[B81] Pratt W. B. Toft D. O. (2003). Regulation of signaling protein function and trafficking by the hsp90/hsp70-based chaperone machinery. *Exp. Biol. Med.* 228 111–133. 10.1177/153537020322800201 12563018

[B82] Prinsloo E. Setati M. M. Longshaw V. M. Blatch G. L. (2009). Chaperoning stem cells: a role for heat shock proteins in the modulation of stem cell self-renewal and differentiation? *BioEssays* 31 370–377. 10.1002/bies.200800158 19274656

[B83] Robert F. (2017). Spt6 gets in the way of polycomb to promote ESC pluripotency. *Mol. Cell* 68 263–264. 10.1016/j.molcel.2017.10.005 29053954

[B84] Rocchi P. Beraldi E. Ettinger S. Fazli L. Vessella R. L. Nelson C. (2005). Increased Hsp27 after androgen ablation facilitates androgen-independent progression in prostate cancer via signal transducers and activators of transcription 3-mediated suppression of apoptosis. *Cancer Res.* 65 11083–11093. 10.1158/0008-5472.CAN-05-1840 16322258

[B85] Rodriguez R. T. Velkey J. M. Lutzko C. Seerke R. Kohn D. B. O’Shea K. S. (2008). Manipulation of OCT4 levels in human embryonic stem cells results in induction of differential cell types. *Exp. Biol. Med.* 232 1368–1380. 10.3181/0703-rm-63 17959850

[B86] Rosenfeld G. E. Mercer E. J. Mason C. E. Evans T. (2013). Small heat shock proteins Hspb7 and Hspb12 regulate early steps of cardiac morphogenesis. *Dev. Biol.* 381 389–400. 10.1016/j.ydbio.2013.06.025 23850773 PMC3777613

[B87] Saibil H. (2013). Chaperone machines for protein folding, unfolding and disaggregation. *Nat. Rev. Mol. Cell Biol.* 14 630–642. 10.1038/nrm3658 24026055 PMC4340576

[B88] Saretzki G. (2004). Stress defense in murine embryonic stem cells is superior to that of various differentiated murine cells. *Stem Cells* 22 962–971. 10.1634/stemcells.22-6-962 15536187

[B89] Saretzki G. Walter T. Atkinson S. Passos J. F. Bareth B. Keith W. N. (2007). Downregulation of multiple stress defense mechanisms during differentiation of human embryonic stem cells. *Stem Cells* 26 455–464. 10.1634/stemcells.2007-0628 18055443

[B90] Scambler P. J. Joseph S. Syed K. M. Dutta D. Majumder A. (2015). Histone chaperone HIRA in regulation of transcription factor RUNX1. *J. Biol. Chem.* 290 13053–13063. 10.1074/jbc.m114.615492 25847244 PMC4505562

[B91] Schlesinger S. Kaffe B. Melcer S. Aguilera J. D. Sivaraman D. M. Kaplan T. (2017). A hyperdynamic H3.3 nucleosome marks promoter regions in pluripotent embryonic stem cells. *Nucleic Acids Res.* 45 12181–12194. 10.1093/nar/gkx817 29036702 PMC5716099

[B92] Schubert U. Antón L. C. Gibbs J. Norbury C. C. Yewdell J. W. Bennink J. R. (2000). Rapid degradation of a large fraction of newly synthesized proteins by proteasomes. *Nature* 404 770–774. 10.1038/35008096 10783891

[B93] Seo N. H. Lee E. H. Seo J. H. Song H. R. Han M. K. (2018). HSP60 is required for stemness and proper differentiation of mouse embryonic stem cells. *Exp. Mol. Med.* 50 e459. 10.1038/emm.2017.299 29546877 PMC5898897

[B94] Setati M. M. Prinsloo E. Longshaw V. M. Murray P. A. Edgar D. H. Blatch G. L. (2010). Leukemia inhibitory factor promotes Hsp90 association with STAT3 in mouse embryonic stem cells. *IUBMB Life* 62 61–66. 10.1002/iub.283 20014282

[B95] Shaik S. Hayes D. Gimble J. Devireddy R. (2017). Inducing heat shock proteins enhances the stemness of frozen–thawed adipose tissue-derived stem cells. *Stem Cells Dev.* 26 608–616. 10.1089/scd.2016.0289 28052723 PMC5393415

[B96] Shakya A. Callister C. Goren A. Yosef N. Garg N. Khoddami V. (2015). Pluripotency transcription factor oct4 mediates stepwise nucleosome demethylation and depletion. *Mol. Cell. Biol.* 35 1014–1025. 10.1128/mcb.01105-14 25582194 PMC4333097

[B97] Shen Z. Formosa T. Tantin D. (2018). FACT inhibition blocks induction but not maintenance of pluripotency. *Stem Cells Dev.* 27 1693–1701. 10.1089/scd.2018.0150 30319048 PMC6302925

[B98] Shinagawa T. Takagi T. Tsukamoto D. Tomaru C. Huynh L. M. Sivaraman P. (2014). Histone variants enriched in oocytes enhance reprogramming to induced pluripotent stem cells. *Cell Stem Cell* 14 217–227. 10.1016/j.stem.2013.12.015 24506885

[B99] Shochet G. E. Komemi O. Sadeh-Mestechkin D. Pomeranz M. Fishman A. Drucker L. (2016). Heat shock protein-27 (HSP27) regulates STAT3 and eIF4G levels in first trimester human placenta. *J. Mol. Histol.* 47 555–563. 10.1007/s10735-016-9699-7 27714564

[B100] Smith A. (2017). Formative pluripotency: the executive phase in a developmental continuum. *Development* 144 365–373. 10.1242/dev.142679 28143843 PMC5430734

[B101] Son Y. S. Park J. H. Kang Y. K. Park J.-S. Choi H. S. Lim J. Y. (2005). Heat shock 70-kDa protein 8 isoform 1 is expressed on the surface of human embryonic stem cells and downregulated upon differentiation. *Stem Cells* 23 1502–1513. 10.1634/stemcells.2004-0307 16100000

[B102] Stewart C. L. Kaspar P. Brunet L. J. Bhatt H. Gadi I. Köntgen F. (1992). Blastocyst implantation depends on maternal expression of leukaemia inhibitory factor. *Nature* 359 76–79. 10.1038/359076a0 1522892

[B103] Strahl B. D. Allis C. D. (2000). The language of covalent histone modifications. *Nature* 403 41–45. 10.1038/47412 10638745

[B104] Takahashi K. Yamanaka S. (2006). Induction of pluripotent stem cells from mouse embryonic and adult fibroblast cultures by defined factors. *Cell* 126 663–676. 10.1016/j.cell.2006.07.024 16904174

[B105] Tan Y. Xue Y. Song C. Grunstein M. (2013). Acetylated histone H3K56 interacts with Oct4 to promote mouse embryonic stem cell pluripotency. *Proc. Natl. Acad. Sci.* 110 11493–11498. 10.1073/pnas.1309914110 23798425 PMC3710873

[B106] Tessarz P. Kouzarides T. (2014). Histone core modifications regulating nucleosome structure and dynamics. *Nat. Rev. Mol. Cell Biol.* 15 703–708. 10.1038/nrm3890 25315270

[B107] Thuret J.-Y. Amigues B. Mann C. Agez M. Guerois R. Lautrette A. (2005). Structural basis for the interaction of Asf1 with histone H3 and its functional implications. *Proc. Natl. Acad. Sci.* 102 5975–5980. 10.1073/pnas.0500149102 15840725 PMC1087920

[B108] Torres-Padilla M.-E. Chambers I. (2014). Transcription factor heterogeneity in pluripotent stem cells: a stochastic advantage. *Development* 141 2173–2181. 10.1242/dev.102624 24866112

[B109] Trusler O. Huang Z. Goodwin J. Laslett A. L. (2018). Cell surface markers for the identification and study of human naive pluripotent stem cells. *Stem Cell Res.* 26 36–43. 10.1016/j.scr.2017.11.017 29227830

[B110] Tsai Y. P. Teng S. C. Wu K. J. (2008). Direct regulation of HSP60 expression by c-MYC induces transformation. *FEBS Lett.* 582 4083–4088. 10.1016/j.febslet.2008.11.004 19022255

[B111] Vilchez D. Boyer L. Morantte I. Lutz M. Merkwirth C. Joyce D. (2012). Increased proteasome activity in human embryonic stem cells is regulated by PSMD11. *Nature* 489 304–308. 10.1038/nature11468 22972301 PMC5215918

[B112] Wadhwa R. Takano S. Kaur K. Aida S. Yaguchi T. Kaul Z. (2006). Identification and characterization of molecular interactions between mortalin/mtHsp70 and HSP60. *Biochem. J.* 391(Pt 2) 185–190. 10.1042/bj20050861 15957980 PMC1276915

[B113] Wang J. Rao S. Chu J. Shen X. Levasseur D. N. Theunissen T. W. (2006). A protein interaction network for pluripotency of embryonic stem cells. *Nature* 444 364–368. 10.1038/nature05284 17093407

[B114] Wang S. Y. Gudas L. J. (1988). Protein synthesis inhibitors prevent the induction of laminin B1, collagen IV (α1), and other differentiation-specific mRNAs by retinoic acid in F9 teratocarcinoma. *cells. J. Cell. Physiol.* 136 305–311. 10.1002/jcp.1041360213 2842348

[B115] Wang Z. Kang L. Zhang H. Huang Y. Fang L. Li M. (2019). AKT drives SOX2 overexpression and cancer cell stemness in esophageal cancer by protecting SOX2 from UBR5-mediated degradation. *Oncogene* 38 5250–5264. 10.1038/s41388-019-0790-x 30894683

[B116] Wong M. M. Yin X. Potter C. Yu B. Cai H. Di Bernardini E. (2014). Over-expression of HSP47 augments mouse embryonic stem cell smooth muscle differentiation and chemotaxis. *PLoS One* 9:e86118. 10.1371/journal.pone.0086118 24454956 PMC3894195

[B117] Wu T. Mu Y. Bogomolovas J. Fang X. Veevers J. Nowak R. B. (2017). HSPB7 is indispensable for heart development by modulating actin filament assembly. *Proc. Natl. Acad. Sci.* 114 11956–11961. 10.1073/pnas.1713763114 29078393 PMC5692592

[B118] Xu H. Wang W. Li C. Yu H. Yang A. Wang B. (2009). WWP2 promotes degradation of transcription factor OCT4 in human embryonic stem cells. *Cell Res.* 19 561–573. 10.1038/cr.2009.31 19274063

[B119] Xu H. M. Liao B. Zhang Q. J. Wang B. B. Li H. Zhong X. M. (2004). Wwp2, An E3 ubiquitin ligase that targets transcription factor Oct-4 for ubiquitination. *J. Biol. Chem.* 279 23495–23503. 10.1074/jbc.M400516200 15047715

[B120] Yan F. Q. Wang J. Q. Tsai Y. P. Wu K. J. (2015). HSP60 overexpression increases the protein levels of the p110α subunit of phosphoinositide 3-kinase and c-Myc. *Clin. Exp. Pharmacol. Physiol.* 42 1092–1097. 10.1111/1440-1681.12457 26174078

[B121] Yan Z. Wei H. Ren C. Yuan S. Fu H. Lv Y. (2015). Gene expression of Hsps in normal and abnormal embryonic development of mouse hindlimbs. *Hum. Exp. Toxicol.* 34 563–574. 10.1177/0960327114555927 25352652

[B122] Yankulov K. (2015). Totipotency in the absence of CAF-I: unhindered choices when the chaperone is out. *Nucleus* 6 468–470. 10.1080/19491034.2015.1121355 26710126 PMC4915508

[B123] Yu H. Lim H. H. Tjokro N. O. Sathiyanathan P. Natarajan S. Chew T. W. (2014). Chaperoning HMGA2 protein protects stalled replication forks in stem and cancer cells. *Cell Rep.* 6 684–697. 10.1016/j.celrep.2014.01.014 24508460

[B124] Zafarana G. Avery S. R. Avery K. Moore H. D. Andrews P. W. (2009). Specific knockdown of OCT4 in human embryonic stem cells by inducible short hairpin RNA interference. *Stem Cells* 27 776–782. 10.1002/stem.5 19350677 PMC2847189

[B125] Zhang B. Peñagaricano F. Driver A. Chen H. Khatib H. (2011). Differential expression of heat shock protein genes and their splice variants in bovine preimplantation embryos. *J. Dairy Sci.* 94 4174–4182. 10.3168/jds.2010-4137 21787952

[B126] Zhang W. Ni P. Mou C. Zhang Y. Guo H. Zhao T. (2016). Cops2 promotes pluripotency maintenance by stabilizing nanog protein and repressing transcription. *Sci. Rep.* 6 4–12. 10.1038/srep26804 27226076 PMC4881025

[B127] Zhang Z. Liao B. Xu M. Jin Y. (2007). Post-translational modification of POU domain transcription factor Oct-4 by SUMO-1. *FASEB J.* 21 3042–3051. 10.1096/fj.06-6914com 17496161

[B128] Zhu Y. Zhu J. Wan X. Zhu Y. Zhang T. (2010). Gene expression of sHsps, Hsp40 and Hsp60 families in normal and abnormal embryonic development of mouse forelimbs. *Toxicol. Lett.* 193 242–251. 10.1016/j.toxlet.2010.01.016 20117194

[B129] Zhu Z. Li C. Zeng Y. Ding J. Qu Z. Gu J. (2017). PHB associates with the hira complex to control an epigenetic-metabolic circuit in human ESCs. *Cell Stem Cell* 20 274.e7–289.e7. 10.1016/j.stem.2016.11.002 27939217

